# A Trajectory-Based Coverage Assessment Approach for Universal Sensor Networks

**DOI:** 10.3390/s150819649

**Published:** 2015-08-11

**Authors:** Ningning Qin, Xin Zheng, Guiyun Tian

**Affiliations:** 1 Key Laboratory of Advanced Process Control for Light Industry Ministry of Education, College of IOT Engineering, Jiangnan University, Wuxi 214122, China; E-Mail: qinnn@jiangnan.edu.cn; 2 School of Electrical and Electronic Engineering, Newcastle University, Newcastle Upon Tyne NE1 7RU, UK; E-Mail: g.y.tian@newcastle.ac.uk

**Keywords:** coverage assessment, universal sensor network, heterogeneous sensor network, homogeneous sensor network, trajectory

## Abstract

To solve the problem of coverage performance assessment, this study proposes an evaluation method based on the trajectory of the target, which is applicable to universal sensor networks, including both heterogeneous and homogeneous sensor networks. Different from the traditional Voronoi algorithm, the proposed Improved Coverage Force Division (ICFD) plans a coverage force division map whichscales the qualitative coverage performancebasedon both covering intensities andlocations of the nodes. Furthermore, the Trajectory-based Evaluating Schedule (TES) is responsible for solving the quantitative coverage evaluationproblem by measuringthe resulting trajectories’ Balance Values (BVs). A model of weak-point ranking conjoined in consideration of coverage force and distance can guide future deployment to compensate coverage. Comparative trials using the greedy algorithm, Voronoi algorithm, and the proposed TES verify that TES achieves the approximate results for two-stage and multistage heterogeneous sensor networks with acceptable difference and lower complexity, and it is superior to the Voronoi algorithm in homogeneous sensor networks interms of breaking the four-point circle block.

## 1. Introduction

With widespread applications of wireless sensor networks [1], it is well known that nodes perform various tasks, such as detection, process, and communication. Sometimes, more than one job must be assigned to some particular nodes. Thus, nodes hardly have the same abilities [2] when fulfilling tasks. Working conditions affect the abilities of the nodes; consequently, their energy affects perception precision, communication distance, and lifetime. Thus, given the influences of physical and environmental factors, nodes cannot be guaranteed to hold exactly the same properties even if they have the same tasks. These non-uniformnodes constitute a type of universal and remarkablenetwork model, named a heterogeneous sensor network [3,4,5].

Clearly, heterogeneity has become a general phenomenon in sensor networks. Many existing outstanding results [6,7,8,9] based on homogeneous sensor networks have been unable to deal with several challenges caused by heterogeneous sensor networks. Therefore, research on heterogeneous sensor networks is important [3,4,5]. As the basis for quality services, heterogeneous sensor networks also haveproblems with coverage evaluation and improvement, which is just similar to normal homogeneous networks. Users should know whether a given sensor network is qualified for certain tasks. Heterogeneous sensor networks are difficult to distribute evenly [6,7] in such a way that all arbitrary nodes can achieve full coverage without any central guidance. Blind and weak spots frequently occur in the monitoring area, which decrease network service quality. 

Therefore, an effective quantitative coverage measure method should be implemented to improve coverage performance and mend weak areas, as well as to provide a comprehensive and accurate evaluation report for the network. As a type of non-uniform network, the heterogeneous sensor network should follow the said method without exception.

Several issues to solve the coverage assessment problem should be considered. Particularly, the questions to be asked are as follows:

(1) How is the quality and the quantity of coverage for universal sensor networks, including homogenous and heterogeneous sensor networks, verified?

(2) How can the overdependence on the whole network information be decreased?

(3) How should future deployment be instructed?

Generally, when coverage performance is assessed, significant attention focuses on the effectiveness of the sensor network standing by the sensor nodes. It is likely that views on the assessment of coverage performance can be changed. If an intruding target is able to easily move through the monitored region, the network is regarded as having inferior coverage quality. Conversely, a network is considered to have better coverage performance if the moving target can be detected with high probability. This new view necessitates a new method to evaluate coverage. As a unique advantage, our study focuses on an intruding moving target of which the moving trajectories are measured through various types of sensor networks to identify the network that has better coverage. To solve the problem, our idea can be described as follows: 

Given a region monitored by a certain sensor network, one fixed starting point, one fixed destination, and one virtual movable target, our study identifies the right moving trajectory, where “right” means minimal detected probability, or the shortest length and other requirements based on the applications. Obviously, the moving trajectories should be different from the various networks although with the same starting point and destination. For example, a low detected probability of a trajectory means better coverage performance of the network. Thus, the difference among trajectories can indicate various coverage qualities. 

In our coverage study, we focus on the minimal accumulation of detected probability on a trajectory.Besides coverage judgment, the presented trajectory-based method can also be good for intrusion detections to preventnetworks against invasion by means of its prediction of the intruding trajectory, especially forsurveillance in military scenarios [8,9]. Under coverage guidance lighted by our assessment results, the sensor network can better serve for other jobs includingvehicle detection [10,11]. Our research accomplishes several tasks and makes the following contributions:

(1) Unfreezing the reliant requirement for global network data. This study presents Improved Coverage Force Division (ICFD) to extend the present local Coverage Force Algorithm (simply as CFA) [12] by proposing the coverage force division map on the basis of absolute coordinates. As a qualitative research result, this map can find the weak sensing points (simply called Weak Points or WPs) without *a priori* knowledge of the overall network topology.
(2) A novel force overlay model of sensing is defined to calculate the sensing force on WPs received from surrounding heterogeneous or homogenous nodes. All WPs are sorted based on this model. Furthermore, the second deployment will be arranged to promote coverage guided by the sorted array of WPs in the future.
(3) Finding the network topology with the best coverage performance is a practical problem in more than one network topology. A Trajectory-based Evaluating Schedule (TES) is designed to evaluate the coverage performance quantitatively, which can help users realize the network that can detect targets effectively among many network distributions.

The study is organized as follows. Section 2 investigates related works and beneficial results. Section 3 includes several valuable notions, models, and assumptions. Section 4 proposes ICFD that can scale the qualitative coverage quality for the universal sensor network and TES, which is responsible for solving the quantitative evaluation problem. Section 5 evaluates the proposed model and schemes by extensive simulations. Section 6 concludes the study.

## 2. Related Works

Numerous research works [13,14,15] have investigated the sensor network coverage performance in terms of various measurement scales, such as point coverage,-coverage [13], coverage holes [14], regional coverage, and target path coverage [15]. Although these methods are effective, most of them must be supported by the overall network information, which is a demanding task and a heavy burden for wireless sensor networks with limited resources. 

**Figure 1:**
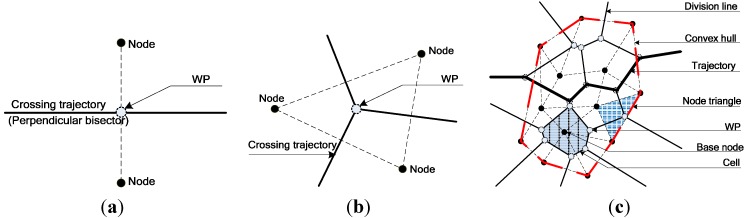
Coverage force division for different uniform nodes. (**a**) Two nodes; (**b**) Three nodes; (**c**) More than three nodes.

The Voronoi diagram [15] algorithmwas an indirect assessment of regional coverage performance although its original objective was to solve the best coverage problem. Its theoretical foundation was that the perpendicular bisector of the two nodes was a crossing trajectory with the weakest coverage force. For three adjacent nodes, three perpendicular bisectors met at the center of gravity, which was obviously the Weak Coverage Point (WP) within the node triangle. For n>3 nodes, perpendicular bisectors and WPs were repeatedly drawn in each node triangle until a Voronoi that has n cells (sub-regions) was built. Figure 1 shows cases with two, three, and more nodes. As shown in Figure 1c, considering that any point within the sub-region is not farther from its base node than the other nodes, they cannot receive less coverage force from its base node than theothers. The judgment, that boundaries of cells are the division lines where coverage forces are weaker than beyond, can be achieved. So, Voronoican apply our trajectory-based idea to evaluate the network performance and will be consideredin the later simulation analysis. However, the algorithm was not strongly recommended because of its limited estimation of coverage quality based on its distribution, whether sparse or not. Moreover, as a type of qualitative evaluation method, the Voronoi algorithm lacked quantitative details and universality. Simultaneously, the Voronoi algorithm wasunavailable for the heterogeneous network and specific topology of a four-point circle (*i.e*., four points lying on the same circle).

Voronoi was employed to detect coverage holes [14] and coverage optimization [16] considering that the vertex of the cell was farthest from the base node. However, these methods could only roughly measure and did not provide sufficient details of the coverage quality. 

In addition to computational geometry such as Voronoi, some concepts in algebraic topology, such as complex and homology theory, were also included in coverage assessment. These concepts are good at rapid detection of coverage holes [17,18] and optimization compensation [19]. Furthermore, compared with the rigorous Ĉech complex that has difficulty in satisfying its application conditions, the Rips complex was verified to have a missing rate problem that could be mended in coverage hole detection by adjusting the ratio between the communication radius and the sensing radius [20].

In terms of the exposure path as a scale, [21] designed a method of measurement by monitoring the moving track of an unauthorized accessing target in a given area. The target trajectory measurement opened a new prospect in studying coverage assessment, although its original focus wasto solve the largest detection interval but to evaluate coverage and improve network performance. Subsequently, Amaldi [22] optimized node positions and analyzed the track exposure on the basis of the detected probability for a moving target. In addition, [14] demonstrated a greedy algorithm which divided a region into approximately infinitegrids based on multiple directions. Based on its division graph, an approximate optimal trajectory with minimal exposure could be found.Although its huge computation is not satisfied, its resulting trajectory canbe taken as the criterion to scale the results of other algorithms in simulations that follow.

Noticeably, the aforementioned methods are constrained within uniform sensor networks. The results which are directly pulled into the heterogeneous networks are not sufficient. Moreover, many existing studies were embodied by the hybrid sensor network [23], which meant that only two types of node would be contained. These were two-stage heterogeneous sensor networks with nodes that have strong or weak abilities [24], or were movable or static [4,23,25], or audio or video [26]. For the three-stage heterogeneous network, Wang [27] set three types of heterogeneous nodes based on the three dimensions of the directional sensor to the optimal deployment. Gupta [28] assumed not more than types of nodes and analyzed the redundancy coverage problem for the heterogeneous network. If the type of node in the network was not among the three types, several existing intelligent algorithms usually became the most popular solutions. Li [29] usedsimulated annealing (SA) and differential evolution to discuss optimal coverage for the heterogeneous network. Genetic optimization [30], cloning [31], swarm intelligence [32], and biological inspiration [33] were also applied to heterogeneous network applications. However, the hardware requirements of these approaches in processing and storage far exceeded the node in terms of applications. 

To the best of our knowledge, these algorithms have strong requirements in global information. However, a practical desirable algorithm should depend only on a small amount of local information. Thus, effectively evaluating the coverage performance without the support of whole network data is an urgent issue that drives our research.

## 3. System Model

### 3.1. Trajectory-Based Idea

Two issues are included in solving the trajectory-based coverage assessment problem. The first issue is how to plan the sensing/coverage force boundaries or the coverage force division, and the second is how to design the right trajectory. The former transforms the huntingzonefrom the infinite plane area into the finite dividing line segment set. The latter is about defining a convenient scale and arranging a schedule to search and assess trajectories.

In terms of the first problem, most of the methods for dividing were inspired by computation geometry, such as the Voronoi algorithm [15,34], greedy algorithm [15,34], and coverage force algorithm (CFA) [12] presented in our early studies, which have been able to partition an area into cells for every node, where all the points can receivemore sensing force from the base node inside than the others outside. These dividing methods fulfill the overall qualitative descriptions for the different kinds of sensor networks. Obviously, these borderlines of adjacent cells are the sensing force boundaries among the nodes. According to the union of all nodes that have sensing capabilities denoting coverage performance, these borderlines were later redefined as coverage force boundaries with no ambiguities.

Given the weights of the boundary segments, understanding that the trajectory problem is the minimal weight route problem is easy. The second problem is solved on the basis of the weighted path table. Many existing and worthwhile routing methods can strongly support our present study. The accumulated weight of the trajectory is a quantitative assessment result.

### 3.2. Network Model

The n static sensor nodes $S=\{s_{i}|i=1,2,\cdots ,n\}$ tasked to detect the moving target T are distributed randomly and independently in the given 2D plane area ${Ι}^{2}$. The sensing parameter $\lambda_{i}$ is identified as the sensing capability of each node $s_{i}$ and $\Lambda =\{\lambda_{i}|i=1,2,\cdots ,n\}$, where $\lambda_{i}>0$. Assuming that a linear relationship exists between the sensing ability and the sensing radius and r is regarded as one coverageradius unit,the sensing/coverage radius of nodes $s_{i}$ can be mapped into $r_{i}=\lambda_{i}\times r$. 

Nodes, whichare able to probe object independently, are be assumed toobtain their distances and calculatepositions through GPS [23], directional antennas technology [28] or others recommended technologies [16,17,19,20,22,26]. Just like many researches [1,2,3,4,5,6,7,8,9], given the position of T, its Euclidean distance to $s_{i}$ $dis_{i}=dis(s_{i},T)$ is regarded as an important adjustment factor in the detection/sensing job. The order-asymptotic sensing model (O-ASM) [35] $Sen\left(s_{i},T\right)={\lambda_{i}/dis_{i}}^{k}$ is used to show nodes’ sensing fluctuation with distanceto T, where integer k is an adjustment parameter for distance, usually *k* ≥ 2.

### 3.3. Exposure Model

To measure the quantitative value, the coverage force amount should be calculated during the continuous moving process of the target. According to the concept of exposure [15], the integral formulation from the original temporal space into the special space [34] can be transformed. The resulting line integral helps to achieve an evaluable coverage force amount for the trajectory, which is a critical foundation for comparison of many trajectories in different scenarios.

During the period of $\left(t_{b},t_{b+1}\right)$, $b\in {\text{Z}}^{+}$, target T moves at a constant speed from position $pos\left(t_{b}\right)$ to position $pos\left(t_{b+1}\right)$ along arbitrary p(t)=(x(t),y(t)) in ${Ι}^{2}$, here $t\in (t_{b},t_{b+1})$, ${\text{Z}}^{+}$ is a positive integer set. If the length of p(t) is L=length(p(t)) and the $n_{i}$ node is present to be involved in the detection in subset $S_{i}$, then ${s_{k}}^{i}\in S_{i}\subseteq S$. The exposure of p(t) can be expressed as follows:

(1) $$Exposure(p(t),S_{i})=\int_{L}\sum_{k}^{n_{i}}Sen({s_{k}}^{i},T)dl,k=1,2,\cdots ,n_{i}$$

Equation (1) shows that the updated expression is only concerned with the length of the track. Exposure is used to indicate the weight. Weight of p(t) is defined as follows:

(2) $$Weight(p(t))=Exposure(p(t),S_{i})$$

### 3.4. Weak Point Queuing

The WP is the position where the sensing resultant force is minimal within a given local sub-region which is outlined by the least amount of nearest nodes and no others inside.

**Figure 2:**
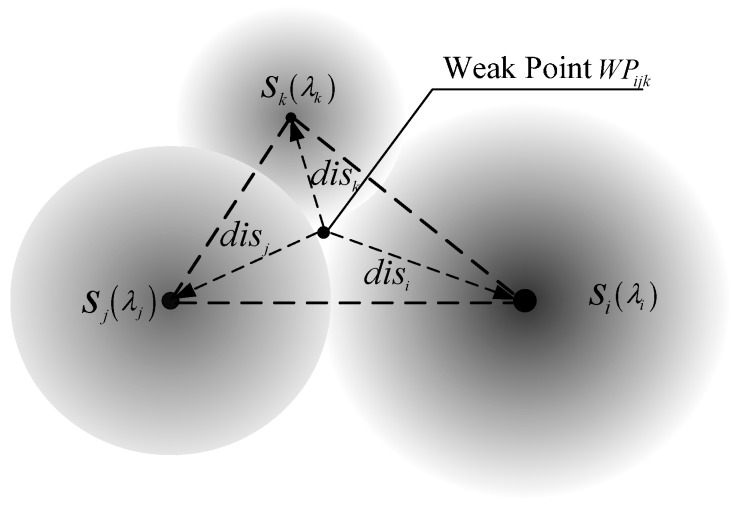
WP within one triangle sub-region, here λ*_i_* > λ*_j_* > λ*_k_*.

If $WP_{ijk}$ is WP within $\Delta s_{i}s_{j}s_{k}$, its Euclidean distances to $s_{i}$, $s_{j}$ and $s_{k}$, respectively are $dis_{i}$, $dis_{j}$ and $dis_{k}$, and their sensing parameters are $\lambda_{i}$, $\lambda_{j}$ and $\lambda_{k}$. Figure 2 shows a triangle sub-region example. We define $Weak_{ijk}$ to present the resultant sensing force for position $WP_{ijk}$, which can be calculatedon the basis of the following Equation:

(3) $$Weak_{ijk}=w_{i}\frac{\lambda_{i}}{di{s_{i}}^{2}}+w_{j}\frac{\lambda_{j}}{di{s_{j}}^{2}}+w_{k}\frac{\lambda_{k}}{di{s_{k}}^{2}}$$

where weights $w_{i}$, $w_{j}$, and $w_{k}$ must satisfy $w_{i}+w_{j}+w_{k}=1$, $0\leq w_{i},w_{j},w_{k}\leq 1$. 

Equation (3) shows that larger $Weak_{ijk}$ means weaker force. $Weak_{ijk}$ is inversely proportional to the distances from nearby nodes and directly proportional to the sensing parameters.

Of course, the resultant sensing force $Weak_{ijk}$ includes the influences of these nodes that join to build a sub-region. Other nodes outside the sub-region are ignored in the study because of the essential influence from distance. They are farther than the ones lying on the hull of the triangle sub-region. Depending on the value of $Weak_{ijk}$, the sorting queue for all the WPs can be achieved. 

If a sub-region is not triangular, Equation (3) should be extended by adding corresponding polymerizations and weights.

### 3.5. Quantitative Model of Trajectory

It is quite possible that notable cases exist where the trajectory takes an elaborate detour to avoid including larger weighted segments. To control the trajectory length, a new Balance Value (simply as BV) is defined as conjoining both the length and the weight, as shown in the following Equation:

(4) $$BalanceValue(p(t),S_{i})=\text{η}\cdot Exposure(p(t),S_{i})+\text{μ}\cdot length(p(t))$$

The balancing coefficients, η and μ are sourced from the different requirements in applications. Theyhave no links to the sensors’ features, but balance the influencing intensities from the exposure and length of trajectory, where 0≤η,μ≤1 and η+μ=1. For example, corresponding todecreasing μ to sacrifice length standard, the value of η is increasedwhich means the detection requirements areenhanced in the assessment process. Without ignoring the various sensing intensities and node distributions, BV can also be defined as the weight for a boundary segment and taken as a scale to measure the network coverage performance. Given the fixed η and μ, greater $BalanceValue(p(t),S_{i})$ indicates better coverage performance. As a commendable virtue, the proposed BV is available for both the uniform homogeneous network and the heterogeneous network.

## 4. Methodology

In our coverage research, without loss of generality, our study maintains the key idea that all different intensities among heterogeneous nodes are considered various sensing magnitude values [12]. In other words, the details of the differences are unimportant compared with the size of the fluctuation that can affect the sensing abilities (coverage forces) of the nodes.Before introducing our proposed ICFD and TES, one important notion and two rules need to be given, where the Division Point (DP) will be defined for the beginning of coverage division, the Conflict Resolution Rule (CRR) will adjust the locations of DPs and the Supplementary Division Rule (SDR) will extend the division results.

### 4.1. Dividing Points

The DPs are composed of Splitting Points (SPs) and WPs. The former is depended on two nodes, and the latter is defined by three nodes.

Given O-ASM, k=2 and two adjacent nodes $s_{i}(x_{i},y_{i})$, $s_{j}(x_{j},y_{j})$ with sensing parameter $\lambda_{i}$, $\lambda_{j}$, an Equipotential Line (shortly EL) exists where sensing forces sourcing from $s_{i}$, $s_{j}$ are equal. The EL can be easily calculated by building the equation for required points based on the sensing forces received from $s_{i}$, $s_{j}$.

If $\lambda_{i}\neq \lambda_{j}$ and $\lambda =\lambda_{i}/\lambda_{j}$, the EL is an Isopotential Circle (shortened as IC), which has a radius of $\sqrt{\lambda }\left|\left(\lambda +1\right)/\left(\lambda -1\right)\right|$ and a center point $\left(x_{centre},y_{centre}\right)$ located on the line of $\bar{s_{i}s_{j}}$, where

(5) $$\begin{array}{c} x_{centre}=\frac{2\lambda (x_{j}-x_{i})}{1-\lambda^{2}}\\ y_{centre}=\frac{2\lambda (y_{j}-y_{i})}{1-\lambda^{2}}+\frac{x_{j}y_{i}-x_{i}y_{j}}{x_{j}-x_{i}} \end{array}$$

The IC and $\bar{s_{i}s_{j}}$ can meet on point SP $SP_{ij}(x_{ij}^{SP},y_{ij}^{SP})$. The location of $SP_{ij}$ can be deduced as shown in the following Equations:

(6) $$\begin{array}{c} x_{ij}^{SP}=\left(x_{j}-x_{i}\right)/\left(\sqrt{\lambda }+1\right)+x_{i}\\ y_{ij}^{SP}=\left(y_{j}-y_{i}\right)/\left(\sqrt{\lambda }+1\right)+y_{i} \end{array}$$

If and only if $\lambda_{i}=\lambda_{j}$, the EL becomes the perpendicular bisector of $\bar{s_{i}s_{j}}$ and its SP is the midpoint of $\bar{s_{i}s_{j}}$. For any point beyond the EL, its coverage forces received from the nearer node is larger than the farther one. In other words, the sensing force of SP is smaller than anywhere of $\bar{s_{i}s_{j}}$. 

On the basis of the WP definition, the objective function to find $WP_{ijk}(x_{ijk}^{WP},y_{ijk}^{WP})$ can be constructed based on the given node triangle $\Delta s_{i}s_{j}s_{k}$, as shown in the following Equation:

(7) $$\begin{array}{l} Min\;\;\;\left|\lambda_{i}dis_{j}^{2}-\lambda_{j}dis_{i}^{2}\right|+\left|\lambda_{k}dis_{j}^{2}-\lambda_{j}dis_{k}^{2}\right|+\left|\lambda_{i}dis_{k}^{2}-\lambda_{k}dis_{i}^{2}\right|\\ \text{S.T.}\;\;\;(x_{ijk}^{WP},y_{ijk}^{WP})\in \Delta s_{i}s_{j}s_{k} \end{array}$$

### 4.2. The Conflict Resolution Rule and the Supplementary Division Rule

Sometimes, SPs and WPs share the same edge. CRR is presented to adjust their positions. A logical explanation for the coexistence of multiple points is that one SP can be calculated based on one certain edge; However, one or two WPs may be conducted separately by two adjacent triangles that share one edge if one of them is an obtuse triangle. 

CRR: If more than one candidate DP (including SPs and WPs) has coexisted on the same edge in one node triangle, the DP with low priority updates its location, and the new location is the same as the one with high priority. The WP is prior to the SP, and a first-come-first-served (FCFS) basis is used for the same priority DP. 

**Figure 3:**
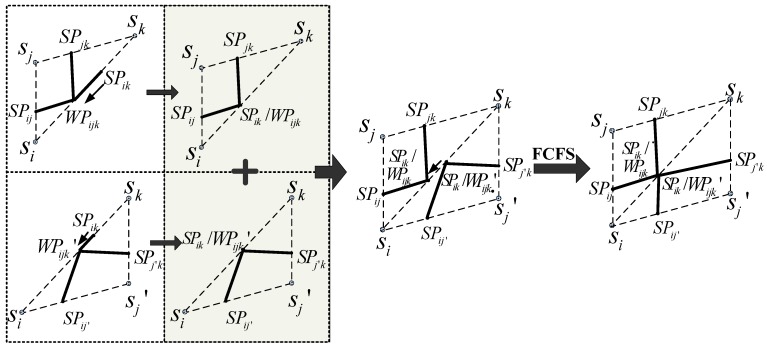
Cases based on CRR where the positions of DPs that share the same edge are updated.

In CRR, the WP hasthe higher prioritybecause of the three nodes calculated, which are more than the two nodes required by SP. Figure 3 presents several desirable cases of CRR. Regardless of the location, four DPs are present and they include one WP and three SPs in one node triangle although they might be located on the same position. All these points are defined as the DPs for the basic division phase. 

However, in the basic division phase, only the division lines can be drawn in the convex hull of the network. Our study supplies SDR to add several division lines between the neighbor nodes on the boundary of the convex hull for the division to be expanded from the inside hull to the entire monitoring region.

**Figure 4:**
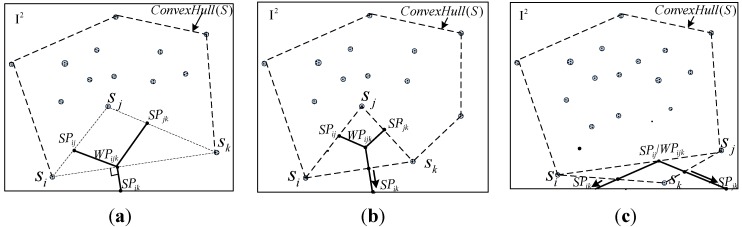
Extension of segment based on SDR (**a**) Two boundarynodes in $\Delta s_{i}s_{j}s_{k}$ and $WP_{ijk}=SP_{ik}$; (**b**) Two boundary nodes in $\Delta s_{i}s_{j}s_{k}$ and $WP_{ijk}\neq SP_{ik}$; (**c**) All three boundary nodes belong to $\Delta s_{i}s_{j}s_{k}$.

SDR: For every boundary edge on the convex hull, two cases have to be considered. For only one DP (regardless of what SP or WP is) located on it, the division line is directly extended outside. Otherwise, a new division line that is perpendicular to the boundary edge is drawn from these DPs to outside (these DPs lie on the same position). The Figure 4 shows the details of the SDR.

### 4.3. Improved Coverage Force Division

Superior to the original CFA [12], the DPs proposed in this study areupdatedusing absolute coordinates. In the obtained division, the division line segments have exhibited that desired feature where the coverage force received from the base node is not less than other nodes. A coverage force division map is generated as follows in Algorithm 1:
**Algorithm 1: ICFD**1-1 Initialization: ${Ι}^{2}$, S, Λ, Starting, Destination, SP=∅, WP=∅, and Edge=∅ 1-2 Δ = Delaunay triangle partition (S) 1-3 For each $\Delta s_{i}s_{j}s_{k}$in set Δ 1-4 Calculate $SP_{ij}$, $SP_{jk}$, $SP_{ik}$ within each pair of adjacent nodes % Using Equation (6) 1-5 Calculate $WP_{ijk}$ within $\Delta s_{i}s_{j}s_{k}$ % Using Equation (7) 1-6 $\left(SP_{ij},SP_{jk},SP_{ik},WP_{ijk})=CRR(SP_{ij},SP_{jk},SP_{ik},WP_{ijk}\right)$ 1-7Update $SP=SP\cup \{SP_{ij},SP_{jk},SP_{ik}\}$ and $WP=WP\cup \{WP_{ijk}\}$ 1-8 Basic Division Phase: Drawing division lines $\bar{SP_{ij}WP_{ijk}}$, $\bar{SP_{jk}WP_{ijk}}$, and $\bar{SP_{ik}WP_{ijk}}$ 1-9 $Edge=Edge\cup \{\bar{SP_{ij}WP_{ijk}},\bar{SP_{jk}WP_{ijk}},\bar{SP_{ik}WP_{ijk}}\}$ -10 End For 1-11 Edge=Edge∪SDR(ConvexHull(S)) 1-12 End ICFD


After the initiation phase, Steps 1–4~1–7 calculate, adjust, and accord DPs’ positions, Steps 1–8~1–9 draw division lines for every cell. And then, the extended edges are added into the Edge set in the last step. These DPs and division lines constitute a coverage force division map.

Our ICFD can partition the region into many cells on the basis of the different coverage forces of the nodes. Unlike the Voronoi algorithm, the ICFD is available for both homogenous and heterogeneous networks.

### 4.4. Trajectory-Based Evaluating Schedule

Inspired by the idea of trajectory-based coverage assessment, we have planned a TES that can produce a quantitative evaluation result. The flow of this TES can be shown as follows in Algorithm 2:
**Algorithm 2: TES**2-1 (Edge,SP,WP)=ICFD(S,Λ) % To build coverage force division map 2-2 For each $WP_{ijk}$ in WP 2-3 Calculate $Weak_{ijk}$ % Using Equation (3) 2-4 End For 2-5 Top_Weak_Point=Sort(WP) 2-6 Edge←Weight(Edge) % Each division line is weighed based on Equation (2) 2-7 Trajectory=Path(Edge,SP,WP)% Using minimal weight path algorithm 2-8 BalanceValue(Trajectory,S) % Using Equation (4) 2-9 End TES


By BalanceValue(Trajectory,S), a judgment on which network has better coverage performance among the various network topologies can be obtained. The returned result Top_Weak_Point identifies the next optimal position for the second deployment.

**Figure 5:**
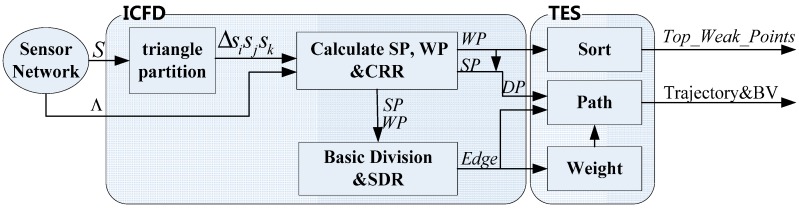
The in-out correlation between the ICFD and the TES.

Regardless of the network being heterogeneous or homogeneous, TES can finish the evaluation effectively, such that the four nodes lying on one circle are no longer obstacles to the evaluation process. Undoubtedly, the aforementioned methods have exciting merits over Voronoi. Furthermore, our schedule can complete the division and valuation tasks without requiring the total network information because the data of only three adjacent nodes are sufficient to drive our schedule. The in-out correlation between the ICFD and the TEShas been shown in Figure 5.

## 5. Simulation Result Analysis

In the Matlab platform, our study simulated several scenarios and problems to validate the ICFD and TES. The public parameter settings are n nodes that are randomly distributed in 2D plane monitoring region ${Ι}^{2}=100\times 100$; the starting point and destination are positioned on the opposite sides outside and the nearest boundary line to enter or exit is selected. The distance parameter is set as k=2 for sensing model O-ASM. For each node, the sensing force is effective for the entire region and the basic sensing radius is $r=150>\left\|{Ι}^{2}\right\|$. $\left\|{Ι}^{2}\right\|$ is the largest spanning distance of the monitoring region. In simulations, the effectivityof trajectory is considered with the same importance as its efficiency. So, η=μ=0.5 is set for BV assessment. Based on the premise of lossless data characteristics, a new relative error model is redefined as $\text{ε}=\mathrm{lg}(\text{δ}\cdot {\text{φ}}^{-1})$, where δ is absolute error and φ is standard error, whereas the result of the greedy algorithm is considered as a reference for comparison. Based on the results of the greedy algorithm, ε can be definedand calculated in order to evaluate other algorithms.

### 5.1. Division, Trajectory and Weak Points

This experiment compares the moving trajectories produced by TES, the greedy algorithm, and Voronoi algorithm, as well as identifies the distribution for WPs. All simulations are repeated in three network scenarios, including homogeneous ($\lambda_{i}=1$), two-stage heterogeneous ($\lambda_{i}\in \{1,2\}$), and multistage heterogeneous ($\lambda_{i}\in [1,3]$). n=20 sensor nodes in the monitoring region.For fair comparison, Equation (2) is also usedfor weighting in the greedy algorithm and Voronoi algorithm, as in our TES.

**Figure 6:**
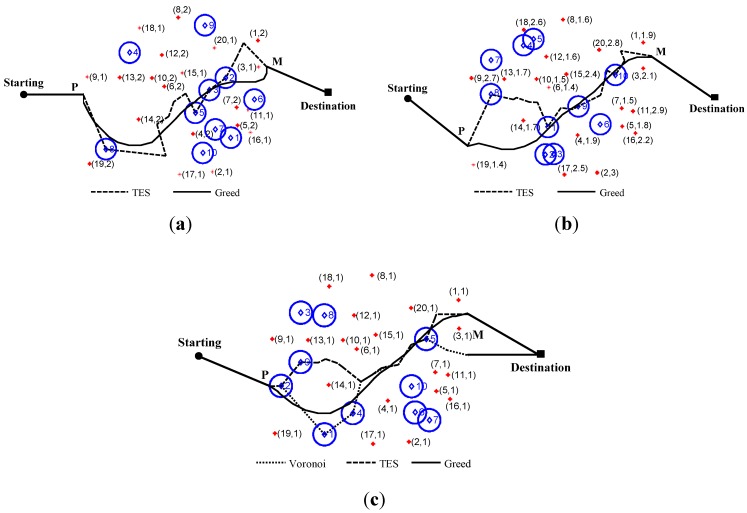
Comparison of trajectories between TES, greedy algorithm, and Voronoi algorithm. (**a**) Two-stage heterogeneous network; (**b**) Multi-stage heterogeneous network; (**c**) Homogeneous network. Where, tag (i, $\lambda_{i}$) nearby node means its label and sensing parameter.

The sensing intensity, resulting trajectories, and WPs are shown in these three scenarios. The top 10 of WPs with tabbed order number are marked as rhombus shapes, which indicate a qualitative analysis of coverage performance. Tags *P* and *M* are the entry and exit points for the trajectory. The trajectory connects the “Starting” point to *P* as the same as “Destination” point to *M* with a straight line because no sensing force is outside the monitoring region. Figure 6a,b shows no results for the Voronoi algorithm because of its limitations. Figure 6c shows Voronoi only in the homogeneous network.

TES tracking trend follows the resulting trajectory of the greedy algorithm with slight differences because the WP, which is one of the smallest sensing force points within the local node triangle, is difficult to match consistently with the one found within the larger region in the greedy algorithm. Similarly, WP has dislocation in the obtuse triangle compared with the Voronoi algorithm because of its barycenter outside the triangle. As a result, all trajectories do not completely coincide even in the homogeneous network.

Compared with the greedy algorithm ($o(|VertexNum_{Greedy}{|}^{3})$$\to o(m^{6})$), TES shows steady and effective working abilities with lower computation load, where $VertexNum_{Greedy}$ is the number of division points in whole monitoring region, andmisthe dividedamountin each edge of unit grid.For TES, the main computing load is calculation of WP with complex $o(n^{3})$, where n is the sampling pointsnumber inside the node triangle. Compared with Voronoiwhich has working limitations, ICFD is devised for both homogeneous and heterogeneous networks. Although differences cannot be avoided in the ICFD method, these differences are acceptable on the basis of error analysis in the next section.

### 5.2. Two-Stage Heterogeneous Network Analysis

In this section, the method is tested for its capability to fulfill coverage assessment in a two-stage heterogeneous network and implement several quantitative analyses on exposure, length, and BV. Given a two-stage random heterogeneous network, the numbers of nodes are set as 15, 20, 25, 30, and 35 with random distribution to show the different node densities. $\lambda_{i}$ is randomly set as 1 or 2. For these five scenarios, the TES and greedy algorithms were independently trialed 30 times, and then all results have been averaged for every scenario. 

**Figure 7:**
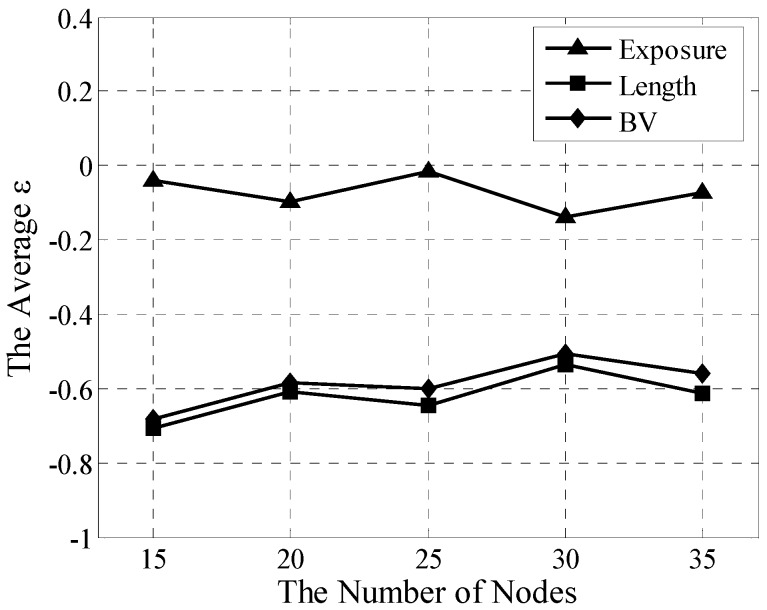
Averaged ε analyses for TES in two-stage heterogeneous network.

Figure 7 displays the averagederrors ε for exposure, length, and BV for TES. As a referenced trajectory, the greedy algorithm obviously does not need to shown ε. Because that the Voronoi can only work in homogeneous network, there aren't any results for Voronoiin this simulation and later simulation about multistage heterogeneous network.With varying density, the averaged ε of ICFD can be stably controlled within [−0.14, −0.01] for exposure, within [−0.72, −0.54] for length, and within [−0.69, −0.55] for BV. Their maximum spans are 0.13, 0.18, and 0.14, which show that ICFD has high stability.

Understandably, because of its smaller path table compared with that of the greedy algorithm, TES is almost unable to find the shorter trajectory. Thus, the longer trajectory of TES can likely lead to closer nodes compared with the greedy algorithm. As exposure is sharply influenced by distance and length, exposure sharply increases with lengthening trajectory in TES, which also explains why the length error is less than the exposure error. Of course, these errors are acceptable because the complexity has been greatly reduced from the TES to greedy algorithm, which not only saves substantial computing resources but also provides a utilitarian method to rapidly assess the coverage performance of the heterogeneous network.

### 5.3. Multistage Heterogeneous Network Analysis

**Figure 8:**
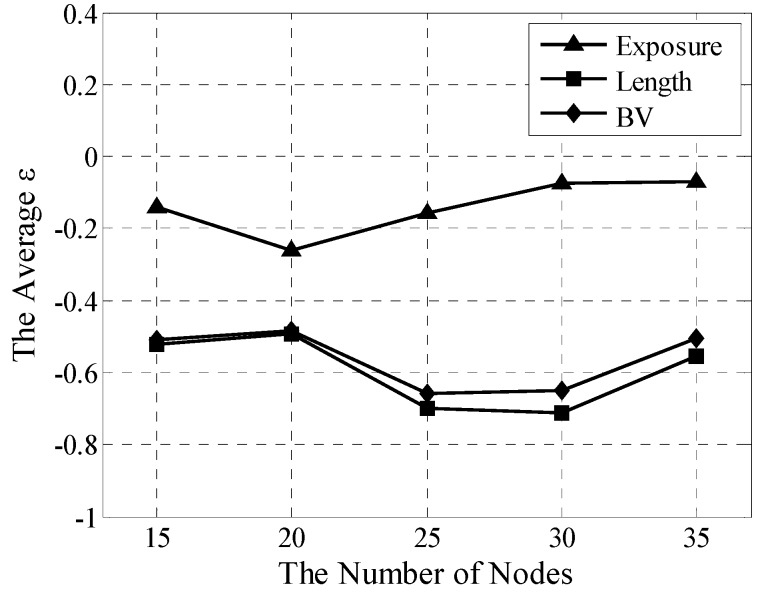
Averaged ε analyses for TES in multistage heterogeneous network.

Widespread multistage heterogeneous networks are used in applications such as surveillance [8,9,12,26] and vehicle detection [10,11]. In this experiment, each $\lambda_{i}$ is set randomly in the range of [0,3]. Other settings and experimental procedures are retained. Error analysis of multistage heterogeneous networks is presented in Figure 8.

Fluctuations in TES are not extremely high and their averaged errors vary within the range of [−0.26, −0.07] for exposure, [−0.73,−0.52] for length, and [−0.64, −0.53] for BV. Its effective and stable operating availabilities are suitable for the quantitative coverage assessment of the multistage heterogeneous network. Moreover, the TESrequires only three neighboring nodes for the algorithm to work. Undoubtedly, its localization can serve as a basis for broad applications in the future. 

### 5.4. Homogeneous Network Analysis

**Figure 9:**
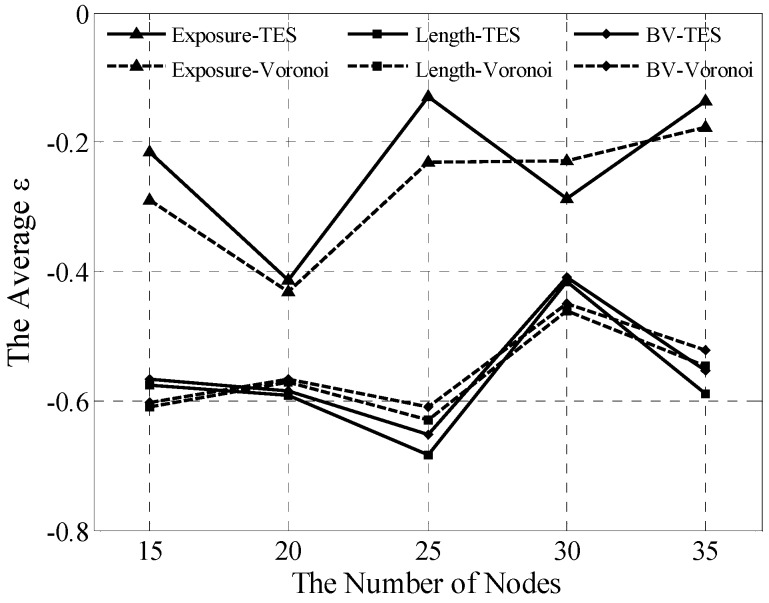
Averaged ε analyses for homogeneous network.

Compared with the Voronoi and greedy algorithms, TES is effective for the homogeneous network, as verified in this section. In simulations, the homogeneous network is easily built by setting $\lambda_{i}=1$. Five scenarios were tested 30 times to achieve the averaged ε. The experimental data are shown in Figure 9. 

Our findings show that TES and Voronoi are not significantly different, which proves that the former can gain results similar to the latter in the homogeneous network. However, existing differences, albeit minimal, must be explained. The different locations of WPs lead to different coverage force divisions even in the same homogeneous network, as mentioned in the preceding section. As shown in Figure 9, the errors should be deemed as an acceptable cost that is offset by desirable values, such as simple, local information requirements and extensive availability.

## 6. Conclusions

In this paper, we develop a localized coverage force division algorithm, ICFD which can providequalitative assessment for coverage quality in a universal sensor network. Moreover, our study presents a quantitative assessment approach, TES to find better coverage performance from various universal sensor networks. Compared with similar assessment methods, ourapproach can serve not only for homogeneous networks but also for heterogeneous networks besides releasing bounds of a four-point circle; and the lower compute load is more suitable for the sensor network. Based on localized coverage force map and trajectory-based assessment, future worksare oriented to solve the more application problems such as optimal deployment and node resource conservation for universal sensor networks.Undeniably, the research for 2D problems is only the beginning. And, assessment problems in 3D will be an interesting challenge. As a bottleneck, energy efficiency will be brought into the trajectory-based assessment in future research.

## References

[1] Karmokar A., Anpalagan A. (2013). Green Computing and Communication Techniques for Future Wireless Systems and Networks. IEEE Potentials.

[2] Samundiswary P., Priyadarshini P., Dananjayan P. Performance evaluation of heterogeneous sensor networks. Proceedings of the 2009 International Conference on Future Computer and Communication (ICFCC2009).

[3] Zhu S., Chen C., Guang X. (2013). Distributed optimal consensus filter for target tracking in heterogeneous sensor networks. IEEE Trans. Cybern..

[4] Wang X., Han S., Wu Y., Wang X. (2013). Coverage and Energy Consumption Control in Mobile Heterogeneous Wireless Sensor Networks. IEEE Trans. Autom. Control.

[5] Tsai R.G., Kuo H.C., Wang H.L., Huang Y.M. Target coverage QoS control with multiple sensing units in wireless heterogeneous sensor networks. Proceedings of the Sixth International Conference on Sensing Technology (ICST’2012).

[6] Wang X., Chen J. (2013). Wireless Sensor Network Node Cover Iterative. Comput. Simul..

[7] Song M., Yang L. (2013). Improving coverage of wireless sensor network using enhanced adaptive PSO algorithm. Appl. Res. Comput..

[8] Vana J., Michele M., Davide B., Vedran B., Benini L. (2014). Benefits of Wake-Up Radio in Energy-Efficient Multimodal Surveillance Wireless Sensor Network. IEEE Sens. J..

[9] Anis K., Khelil A. (2015). Cooperative Robots and Sensor Networks.

[10] Borstell H., Kluth J., Jaeschke M., Plate C., Gebert B., Richter K. Pallet monitoring system based on a heterogeneoussensor network for transparent warehouse processes. Proceedings of the Sensor Data Fusion: Trends, Solutions, Applications (SDF).

[11] Tolga Z., Hwa C. Delay tolerant network for autonomous robotic vehicle charging and hazarddetection. Proceedings of the 2014 IEEE/ACIS 13th International Conference on Computer and Information Science (ICIS).

[12] Qin N., Zhang L., Xu B. (2010). The Coverage force algorithm for heterogeneous wireless sensor networks. J. Electron. Inf. Technol..

[13] Du X., Lin F. (2005). Maintaining differentiated coverage in heterogeneous sensor networks. EURASIP J. Wirel. Commun. Netw..

[14] Babaie S., Pirahesh S.S. (2012). Hole detection for increasing coverage in wireless sensor networks using triangular structure. Int. J. Comput. Sci. Issues.

[15] Meguerdichian S., Koushanfar F., Qu G., Potkonjak M. Exposure in wireless ad hoc sensor networks. Proceedings of the ACM SIGMOBILE Annual International Conference on Mobile Computing and Networking.

[16] Mahboubi H., Moezzi K., Aghdam A.G., Sayrafian-Pour K. Self-deployment algorithms for field coverage in a network of nonidentical mobile sensors: Vertex-based approach. Proceedings of the 2011 American Control Conferenceon O’Farrell Street.

[17] Kanno J., Buchart J.G., Selmic R.R., Phoha V. Detecting coverage holes in wireless sensor networks. Proceedings of the 17th Mediterranean Conference on Control and Automation.

[18] Ghrist R., Muhammad A. Coverage and hole-detection in sensor networks via homology. Proceedings of the 4th International Symposium on Information Processing in Sensor Networks (IPSN’05).

[19] Yan F., Martins P., Decreusefond L. Connectivity-based distributed coverage hole detection in wireless sensor networks. Proceedings of the IEEE Global Telecommunications Conference.

[20] Yan F., Martins P., Decreusefond L. Accuracy of homology based approaches for coverage hole detection in wireless sensor networks. Proceedings of the IEEE International Conference on Communications.

[21] Deng K.B., Liu Z. (2007). Minimum path exposure and detection interval setting for target detection using wireless sensor network. Int. J. Wirel. Inf. Netw..

[22] Amaldi E., Capone A., Cesana M., Filippini I. (2012). Design of wireless sensor networks for mobile target detection. IEEE/ACM Trans. Netw..

[23] Dung T.N., Nam P.N., My T.T., Abdelsalam H. An optimal algorithm for coverage hole healing in hybrid sensor networks. Proceedings of the 7th International Wireless Communications and Mobile Computing Conference (IWCMC).

[24] Lee J., Krishnamachari B., Kuo C. Impact of heterogeneous deployment on lifetime sensing coverage in sensor networks. Proceedings of the First Annual IEEE Communications Society Conference on Sensor and Ad Hoc Communications and Networks.

[25] Liu B., Dousse O., Nain P., Towsley D. (2013). Dynamic coverage of mobile sensor networks. IEEE Trans. Parallel Distrib. Syst..

[26] Kushwaha M. (2010). Feature-Level Information Fusion Methods for Urban Surveillance Using Heterogeneous Sensor Networks. Ph.D. Thesis.

[27] Wang L., Xu Z., Wu X., Cheng H. (2012). Optimal Deployment Scheme in Sensing-heterogeneous Wireless Sensor Networks. J. Nanjing Univ. Sci. Technol..

[28] Gupta H.P., Rao S.V., Venkatesh T. Analysis of the redundancy in coverage of a heterogeneous wireless sensor network. Proceedings of the 2013 IEEE International Conference on Communciation.

[29] Li M. (2011). Study on Coverage Algorithms for Heterogeneous Wireless Sensor Network. Ph.D. Thesis.

[30] Abdusy S., Imee B., Abedlhafid A., Lhassane I., Riri F., Pascal L. Evolutionary multi-objective based approach for wireless sensor network deployment. Proceedings of the IEEE International Conference on Communications (ICC).

[31] Jiang C., Wang H., Chen L., Shi J. (2012). A Novel Security Algorithm Based on Biological Immune Principles in Wireless Sensor Networks. J. Jilin Univ. (Science Edition).

[32] Lee J.W., Lee J.J. (2012). Ant-colony-based scheduling algorithm for energy efficient coverage of WSN. IEEE Sens. J..

[33] Miao L., Qi H., Wang F. Biologically inspiredself deployable heterogeneous mobile sensor networks. Proceedings of the IEEE/RSJ International Conference on Intelligent Robots and Systems (IROS 2005).

[34] Qin N., Zhang L., Shan X., Xu B. (2007). Locomotion trajectory with cooperative metrics in wireless sensor networks. J. China Univ. Posts. Commun..

[35] Qin N., Jiang M., Xiong W., Xu B. (2010). Comparison between the node sensing models and the relative works in wireless sensor networks. Appl. Res. Comput..

